# Use of Machine Learning to Predict the Incidence of Type 2 Diabetes Among Relatively Healthy Adults: A 10-Year Longitudinal Study in Taiwan

**DOI:** 10.3390/diagnostics15010072

**Published:** 2024-12-31

**Authors:** Ying-Qiang Liu, Tzu-Wei Chang, Lung-Chun Lee, Chia-Yu Chen, Pi-Shan Hsu, Yu-Tse Tsan, Chao-Tung Yang, Wei-Min Chu

**Affiliations:** 1 Department of Medical Education, Taichung Veterans General Hospital, Taichung 407219, Taiwan; 2 Department of Family Medicine, Taichung Veterans General Hospital, Taichung 407219, Taiwan; 3 Division of Family Medicine, Department of Medicine, Taipei Veterans General Hospital Yuanshan Branch, Yilan 264018, Taiwan; 4 Department of Application Value-Added Service, SYSTEX Corporation, Taipei 114730, Taiwan; 5 Division of Occupational Medicine, Department of Emergency Medicine, Taichung Veterans General Hospital, Taichung 407219, Taiwan; 6 School of Medicine, Chung Shan Medical University, Taichung 402306, Taiwan; 7 Department of Computer Science, Tunghai University, Taichung 407224, Taiwan; 8 Research Center for Smart Sustainable Circular Economy, Tunghai University, Taichung 407224, Taiwan; 9 Geriatrics and Gerontology Research Center, College of Medicine, National Chung Hsing University, Taichung 402202, Taiwan; 10 Department of Post-Baccalaureate Medicine, College of Medicine, National Chung Hsing University, Taichung 402202, Taiwan; 11 School of Medicine, National Yang Ming Chiao Tung University, Taipei 112304, Taiwan

**Keywords:** machine learning models, diabetes, free thyroxine, glycated hemoglobin, fasting blood glucose, weight, triglycerides

## Abstract

**Background**: The prevalence of diabetes is increasing worldwide, particularly in the Pacific Ocean island nations. Although machine learning (ML) models and data mining approaches have been applied to diabetes research, there was no study utilizing ML models to predict diabetes incidence in Taiwan. We aimed to predict the onset of diabetes in order to raise health awareness, thereby promoting any necessary lifestyle modifications and help mitigate disease burden. **Methods**: The research dataset used in the study was retrieved from the Clinical Data Center of Taichung Veterans General Hospital. We collected data from the available electronic health records with a total of 33 items being employed for model construction. Individuals with diabetes and those with missing data were excluded. Ultimately, 6687 adults were included in the final analysis, where we implemented three different ML algorithms, including logistic regression (LR), random forest (RF) and extreme gradient boosting (XGBoost) in order to predict diabetes. **Results**: The top five important factors involved in the prediction model were glycated hemoglobin (HbA1c), fasting blood glucose, weight, free thyroxine (fT4), and triglycerides (TG). Notably, random forest, logistic regression, and XGBoost reached 99%, 99%, and 98% accuracy, respectively. fT4 seems to be one of the significant features in predicting the onset of diabetes. Moreover, this would be the first study using machine learning models to predict diabetes that has demonstrated the importance of thyroid hormone. **Conclusions**: A total of 33 items were able to be put into the machine learning model in order to predict diabetes with promising accuracy. In comparison to prior studies on machine learning models, this study not only identified similar key factors for predicting diabetes but also highlighted the significance of thyroid hormones, a factor that was previously overlooked. Moreover, it highlighted the relevance of predicting type 2 diabetes using more affordable methods, which would be useful for clinical healthcare professionals and endocrinologists who apply the models to clinical practice.

## 1. Introduction

The prevalence of type 2 diabetes has been increasing throughout the world, reflecting a rising burden on financial expenditures and public health [1]. Remarkably, Pacific Ocean island nations have the highest prevalence of type 2 diabetes. Additionally, the condition is also one of the leading causes of human suffering, affecting quality of life and even premature mortality [2]. Of major concern is that the onset of type 2 diabetes may be delayed 9–12 years upon diagnosis, when patients who present themselves with established microvascular complications, such as diabetic retinopathy, can be treated [3]. It remains important to identify patients with undiagnosed type 2 diabetes during the preclinical period [4], as well as to predict the disease among relatively healthy adults.

For asymptomatic individuals, a conventional medical checkup performed by healthcare professionals involving history taking, physical examinations, and laboratory tests and imaging is essential [5]. Additionally, medical checkups are all part of preventive medicine and provide opportunities for medical personnel to educate the patient while also disseminating information regarding this chronic disease [6], therefore, raising health awareness. Regarding issues surrounding society and government, Christian et al. found that the global cost of diabetes will substantially increase by 2030, greatly impacting the world’s healthcare systems, necessitating that urgent action be taken in order to mitigate its economic burden [7]. In the coming future, reducing the impact that diabetes has on both global health and its economy will remain a challenging issue.

During the onset of this decade, the COVID-19 pandemic raged worldwide, leading to an unprecedented hazardous effect on mental health due to the policies of social distancing and quarantine [8]. Indeed, the virus caused a deterioration in, and a negative impact on, many individual’s mental health [9] and it may have indirectly raised public awareness of mental health partly due to fear caused by the pandemic. Intriguingly, multiple mechanisms have been postulated, which may explain the rising rate of new-onset diabetes in COVID-19 patients. One of these mechanisms is that severe acute respiratory syndrome coronavirus 2 (SARS-CoV-2) enters the islet cell through the angiotensin-converting enzyme-2 (ACE-2) receptors [10], which may eventually impede insulin secretion. A meta-analysis found that 19.70% (95% CI: 10.93–32.91) of COVID-19 patients experienced new-onset diabetes [11]. Given the higher risk of newly detected diabetes in the post-acute COVID-19 population, screening remains essential regardless of the severity during the acute phase of COVID-19 [12]. Through advancing technology, Artificial Intelligence (AI) applications are now being seen in the healthcare system and are gradually receiving attention from healthcare professionals as they become more involved in clinical decision-making [13]. One example is how predicting physical function upon discharge among older adults based upon data taken from electronic health records (EHRs) is now being used [14]. The machine learning (ML) model develops algorithms to analyze patterns from the data taken from EHRs. However, there are still certain limitations due to data quality [15].

To date, numerous ML models and data mining approaches have been applied to diabetes research [16]. Not only the prediction of the onset of diabetes complications but also the early diagnosis of type 2 diabetes can be carried out. Dagliati et al. [17] in Italy discovered that applying data mining methods, as well as using demographic data and clinical data from EHRs among nearly 1000 patients suffering from type 2 diabetes in order to develop predictive models for the onset of microvascular complications was a promising approach. In recently performed research, a novel ML algorithm, called the extreme gradient boosting (XGBoost) classifier, is even applied as a prediction model for type 2 diabetes, where it reaches a high identification rate [18].

However, any relevant literature regarding the use of ML models for predicting diabetes incidence is scarce in the Asia-Pacific region [18,19], and some of that literature was only completed via a questionnaire survey [20]. Therefore, it remains clear that additional research using ML to predict type 2 diabetes among healthy adults is still warranted. Thus, the objectives of this study are:

1. to select appropriate features predicting type 2 diabetes among relatively healthy adults;
2. to establish a prediction model via the use of different ML algorithms and subsequently compare the performance accuracy.

This is the first study utilizing EHR data taken from a tertiary medical center for the purpose of diabetes prediction among healthy adults in Taiwan having a 10-year longitudinal design.

## 2. Materials and Methods

### 2.1. Dataset

The research dataset was retrieved from the Clinical Data Center of Taichung Veterans General Hospital. Relatively healthy adults who had undergone at least two self-paid health examinations within an interval of less than four years during the period from January 2011 to June 2021 were included for analysis. We collected data from EHRs regarding general demographics, medical history, and laboratory tests, with a total of 33 items being employed for model construction. Individuals with a medical history of diabetes prior to the study period and those with missing data were excluded. The parameters used included age, gender, height, weight, waist size, pulse rate, respiration rate, total cholesterol (TC), triglycerides (TG), high density lipoprotein cholesterol (HDL-C), low density lipoprotein cholesterol (LDL-C, measured), aspartate transaminase (AST), alanine transaminase (ALT), total bilirubin, direct bilirubin, r-glutamyl transferase (r-GT), total protein, albumin, blood urea nitrogen (BUN), serum creatinine, estimated glomerular filtration rate (eGFR), uric acid, serum sodium, serum calcium, hemoglobin (Hgb), platelets, high-sensitivity c-reactive protein (hsCRP), fasting blood glucose, glycated hemoglobin (HbA1c), thyroid stimulating hormone (TSH), free thyroxine (fT4), urine glucose, and urine ketone. Ultimately, 6687 adults were included in the final analysis. This study was conducted in accordance with the Declaration of Helsinki and approved by the Institutional Review Board of Taichung Veterans General Hospital (protocol code: TCVGH-IRB CE21445B-1, date of approval: 5 January 2023). The Patient Consent form was waived by TCVGH-IRB due to the study’s retrospective design.

### 2.2. Data Pre-Processing

Initially, we merged EHRs and excluded adults who had been diagnosed with diabetes. Individuals were classified as potential diabetes cases based on an HbA1C > 6.5% or a fasting glucose >126 mg/dL. The annotated data after organization were categorized using the following four labels: “No diabetes symptoms”, “Potential diabetes symptoms”, “Confirmed diabetes”, and “No data”. Meanwhile, the “df.isnull().sum()” method was used to calculate the number of missing values in each column. A DataFrame entitled “Missing” was created, containing column names and the percentage of missing values. Subsequently, a horizontal bar plot depicting the missing ratios was generated, and any columns with missing ratios exceeding 20% were removed. Recognizing that filling in missing values with a specific number may impact the model’s prediction ability, the remaining missing values were completed using “−999” as an indicator that the values were missing values. Finally, the “train_test_split function” from the “scikit-learn version 1.6 (sklearn 1.6)” software machine learning library was used to partition the data into training and testing sets with a split ratio of 60% and 40%. The training set was utilized for model training, while the testing set was employed to evaluate the model’s performance.

### 2.3. Machine Learning and Prediction Model Development

#### 2.3.1. Random Forest (RF)

RF belongs to Ensemble Learning, representing a more sophisticated decision tree. Comprising multiple decision trees, each tree operates independently within the ensemble. In the classification process, every new sample undergoes both evaluation and classification by each decision tree within the forest. Consequently, each decision tree produces an individual classification result, with the Random Forest consolidating the outcomes through a voting mechanism, aggregating the classification results from all the decision trees.

The RF equation is as follows:

(1) $$RF=\sum_{k=1}^{K}P_{k}\left(1-P_{k}\right)=1-\sum_{k=1}^{K}P_{k}^{2}$$

#### 2.3.2. Logistic Regression (LR)

The LR model serves as a form of linear classifier, mainly used in binary classification scenarios. It is primary for classifying data based on the available information and makes judgments to assign data points to specific categories. Notably, the output values generated by the logistic regression model for classification purposes are constrained within the range of [0, 1].

#### 2.3.3. XGBoost

The full name of XGBoost is Extreme Gradient Boosting. This algorithm maintains the integrity of the original model through each iteration and adds a new function to the model. This process enables subsequent trees to correct errors made by preceding trees. Moreover, XGBoost incorporates random feature sampling during tree generation, ensuring that not all features are considered in decision-making for each tree. The algorithm contributes to the model’s diversity and robustness, while also enhancing the predictive performance.

The equation of XGBoost is as follows:

(2) $$XGBoost=\sum_{i=1}^{n}l\left(y_{i},y_{i}^{\left(t\right)}\right)+\sum_{i=1}^{t}\Omega \left(f_{i}\right)$$

### 2.4. Data Analysis Through Machine Learning

Through the aforementioned data pre-processing, we employed various machine learning models during data predictive analysis, including RF, LR, and XGBoost. Furthermore, we used SHAP (Shapley Additive exPlanations) in order to perform a visualization of the model’s attention to data features [21,22]. This could help contribute to enhancing the interpretability of the model prediction as well as enable us to better comprehend the reasons and decision-making processes behind the models. Consequently, the predictive outcomes become more trustworthy and interpretable. Such an approach assists us towards reaching a deeper interpretation of the data, thus allowing us to make more valuable predictions and decisions.

## 3. Results

The final analysis encompassed a total of 6687 adults. Table 1 shows the demographics and clinical characteristics of all participants during their first health exam. The average age was 57.7 years, while the average FBS and HbA1c was 89.6 and 5.5, respectively, suggesting that participants were relatively healthy when they underwent their health exam.

In Table 2, the precision, recall, and F1-score of the classification models were each revealed. The accuracy rates for RF, LR, and XGBoost were 99%, 99%, and 98%, respectively. The precisions for the prediction of normal cases in RF, LR, and XGBoost were all 99%. However, the precisions for diabetes cases in RF, LR, and XGBoost were 25.0%, 0%, and 19.0%, respectively.

**Figure 1:**
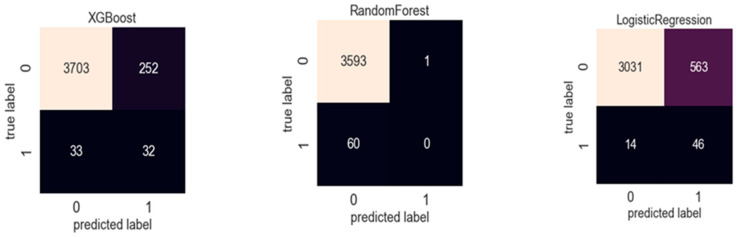
Confusion matrix of machine learning model by XGBoost, Random Forest, and logistic regression.

The confusion matrix of ML in different prediction models is depicted in Figure 1. RF and XGBoost showed relatively good results; however, LR was poor in differentiating true diabetic patients.

**Figure 2:**
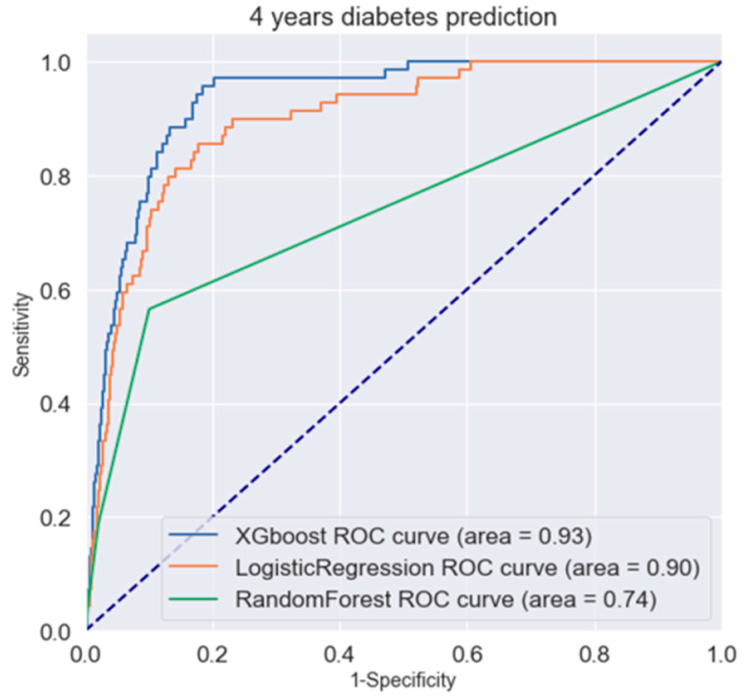
ROC curve of machine learning model by XGBoost, Random Forest, and logistic regression for 4-year diabetes prediction.

Examining the area under the ROC (auROC) curve as the performance metric, in Figure 2 for a 4-year diabetes prediction, both XGBoost and LR models exhibited an auROC exceeding 90%, while RF had an auROC of 74%.

**Figure 3:**
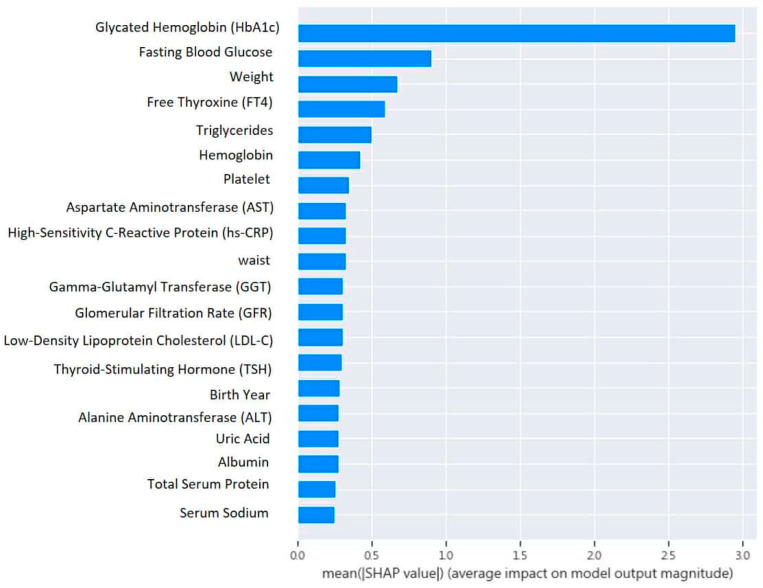
Feature importance of machine learning model by XGBoost predicting incidence of type 2 diabetes among relatively healthy adults.

The classification algorithm by XGBoost successfully identified key features in the process, as illustrated in Figure 3. Notably, the top five features of importance remained consistent: HbA1c, fasting blood glucose, weight, fT4, and TG.

## 4. Discussion

Despite the highest prevalence and disease burden of type 2 diabetes seen in the Pacific Ocean island nations, there still remains a lack of the clinical tools necessary in order to predict diabetes incidence. To the best of our knowledge, this is the first study to utilize ML models and EHRs to predict diabetes incidence among relatively healthy adults in Taiwan. The results demonstrate that diabetes incidence can be accurately predicted through the use of ML models, including LR, RF, and XGBoost, when combined with key clinical features. Our ML models yielded robust predictions for categorical outcomes. Furthermore, the top five features of importance were HbA1c, fasting blood glucose, weight, fT4, and TG. Consequently, we contend that the application of Artificial Intelligence can assist healthcare professionals in recognizing the early stages of diabetes, thereby enhancing its clinical applicability.

In the past, numerous studies have utilized ML models to investigate the onset of diabetes complications; however, there are relatively fewer studies exploring the prediction of any early detection of diabetes. Dagliati et al. [17] applied a logistic regression model with selected features to predict the onset of diabetes complications, including retinopathy, neuropathy, and nephropathy, at different time scenarios among a type 2 diabetes population. Their results showed that data mining methods could be adopted in clinical medicine in order to support clinical practice. Wu et al. [18] used a novel binary logistic regression, the XGBoost classifier, as a prediction model for type 2 diabetes. Additionally, the team made it adaptive to more than one dataset, including both the Pima Indians Diabetes Database (PIDD) and Early-Stage Diabetes Risk Prediction Database (ESDRPD), coming up with a 94% and 98% identification rate for diabetes prediction, respectively. Recently, Abnoosian et al. [23] proposed an innovative multi-classification framework using the imbalanced Iraqi Patient Dataset of Diabetes in order to predict diabetes and found that the model outperformed other machine learning models in diabetes prediction. Additionally, the study also discussed the use of various classification algorithms, data preprocessing techniques, and the impact of different parameters on model accuracy, as other previous research had done [24]. Their study highlighted the potential of how a proposed framework can serve as a valuable tool. From our study, the ML models were able to predict type 2 diabetes accurately using selected clinical features among relatively healthy Asian adults. This is certainly valuable for supporting clinical practice and enhancing people’s health awareness.

### 4.1. Features of Importance

There were several studies utilizing machine learning to predict diabetes [25,26,27]. Fasting blood glucose is one of the significant features in the studies. Although this study revealed a similar result, it also revealed the importance of fT4. The association between thyroid function and diabetes appears to be less intuitive. In clinical settings, thyroid function measurement, including thyroid-stimulating hormone (TSH) and fT4, is not included in routine screenings for diabetes, particularly in people without any apparent symptoms of thyroid dysfunction. However, with regard to the findings of our study, fT4 seems to be one of the significant features in predicting the onset of diabetes. The thyroid hormone plays a role in both regulating metabolism and energy expenditure, while it is also involved in insulin regulation and glucose homeostasis [28]. In accordance with the previous literature, the thyroid hormone has been shown to augment beta-cell viability [29]. Chaker et al. [30] performed a population-based prospective cohort study and discovered that subclinical hypothyroidism is a risk factor for incident diabetes among individuals with prediabetes. Recently, a study conducted in Japan demonstrated that both hyperthyroidism and hypothyroidism correlate with type 2 diabetes, and that a population with subclinical hypothyroidism showed a higher incidence of diabetic complications [31]. Future studies are still warranted in order to better explore whether thyroid function measurement should be incorporated into diabetes screening among relatively healthy adults. Furthermore, the relevance of predicting type 2 diabetes using more affordable methods, such as triglycerides, compared to the cost of the HbA1C test should be explored. This would be useful for clinical healthcare professionals and endocrinologists who apply the models to clinical practice.

### 4.2. Strength and Limitations

The study has some limitations. First, the data investigated came from a single tertiary hospital without validation from any other available public databases, thus external validity should be interpreted cautiously. It remains uncertain whether the models used can be generalized to other healthcare systems. Second, the inclusion of numerous clinical features may pose challenges for smaller-scale healthcare systems which may not be able to collect comprehensive clinical data. This may potentially impact the accuracy of the model. However, our study implemented three different ML algorithms and compared their accuracy, emphasizing the potential of machine learning in predicting diabetes using medical informatics [32]. Moreover, the results highlighted that thyroid function may be a significant factor for diabetes prediction among relatively healthy adults. This would be the first study using machine learning models to demonstrate the importance of thyroid hormone in predicting diabetes.

### 4.3. Implications

Our study indicates that diabetes prediction among relatively healthy adults is feasible using EHRs and ML models. For clinical healthcare professionals involved in preventive medicine, we believe that the prediction model could apply to both clinical practice and shared decision-making, particularly for individuals seeking to raise health awareness, thereby promoting any necessary lifestyle modifications. Diabetes prediction could not only prevent the onset of an irreversible disease, but it may also mitigate the burden of excess financial expenditures, which would negatively impact the healthcare system over the coming years. Additionally, the clinical features highlight the importance of thyroid function as being a significant factor. Due to the promising accuracy resulting from these ML models, we aim to promote the application of the models to other healthcare systems in the near future.

## 5. Conclusions

We were able to predict diabetes among relatively healthy adults through EHRs with regard to general demographics, medical history, and laboratory tests, while involving a total of 33 items. We found that HbA1c, fasting blood glucose, weight, fT4 and TG are the five important factors involved in the prediction model. The accuracy rates for RF, LR, and XGBoost were 99%, 99%, and 98%, respectively. In the future, we will seek to explore the application in different ML algorithms and apply it to the available public databases in order to verify its generality and versatility. Furthermore, this would allow us to make the models more practical for further application in other healthcare systems with regard to their use as a clinical tool.

## Figures and Tables

**Table 1:** Demographic and clinical characteristics of participants.

Parameter	Mean	25%	50%	75%
age	57.7	50	58	65
height (cm)	165.7	159.5	165.9	171.7
weight (kg)	66.2	56.8	65.6	74.0
waist (cm)	80	73	80	86
respiration rate (/min)	16.6	16	16	18
pulse (/min)	70.6	63	70	77
AST (U/L)	24.3	18	22	27
ALT (U/L)	30.1	17	24	35
total bilirubin (mg/dL)	0.88	0.6	0.8	1.0
direct bilirubin (mg/dL)	0.5	0.5	0.5	0.5
r-GT (U/L)	33.9	16	24	36
serum creatinine (mg/dL)	0.84	0.70	0.83	0.99
BUN (mg/dL)	12	10	12	14
eGFR (ml/min/1.73m2)	97.91	83.09	94.74	109.11
FBS (mg/dL)	89.6	84	89	95
HbA1c (%)	5.5	5.3	5.5	5.7
TC (mg/dL)	198.2	174	196	220
TG (mg/dL)	127.5	72	105	155
HDL-C (mg/dL)	55.1	44	53	64
LDL-C, measured (mg/dL)	116.45	96.3	113.0	136.0
Hgb (g/dL)	14.4	13.3	14.5	15.5
platelet (1000/μL)	243.3	203	238	277
albumin (g/dL)	4.5	4.3	4.5	4.7
total protein (g/dL)	7.3	7.0	7.3	7.6
uric acid (mg/dL)	6.2	5.0	6.1	7.2
fT4 (ng/dL)	13.2	11.2	12.3	14.8
hsCRP (mg/L)	0.1688	0.021	0.058	0.158
serum sodium (mEq/L)	143.4	142	143	145
serum calcium (mg/dL)	8.9	8.7	8.9	9.2
urine glucose (mg/dL)	8.8	0	0	0
urine ketone (mg/dL)	0.2	0	0	0

AST, aspartate transaminase; ALT, alanine transaminase; r-GT, r-glutamyl transferase; BUN, blood urea nitrogen; eGFR, estimated glomerular filtration rate; FBS, fasting blood glucose; HbA1c, glycated hemoglobin; TC, total cholesterol; TG, triglycerides; HDL-C, high density lipoprotein cholesterol; LDL-C, low density lipoprotein cholesterol; Hgb, hemoglobin; fT4, free thyroxine; hsCRP, high-sensitivity c-reactive protein.

**Table 2:** Precision, recall, F1-score and accuracy of machine learning model by XGBoost, Random Forest, and logistic regression with normal cases and potential diabetes cases.

	Class 0 (Normal Cases), Support: 6967			Class 1 (Potential Diabetes Cases), Support: 76				
	Precision	Recall	F1-Score	Precision	Recall	F1-Score	Accuracy	Macro F1-Score
Random Forest	0.99	1.00	0.99	0.25	0.01	0.03	0.99	0.51
Logistic regression	0.99	1.00	0.99	0.00	0.00	0.00	0.99	0.50
XGBoost	0.99	0.98	0.99	0.19	0.33	0.24	0.98	0.61

## Data Availability

The datasets generated and analyzed during the study are available from the corresponding author on reasonable request.
